# Relating Domain-Specific Risk-Taking Behavior to Cognitive Functions in Older Adults

**DOI:** 10.3390/brainsci15101044

**Published:** 2025-09-25

**Authors:** Leah H. Waltrip, Silvia Chapman, Madison Bouchard-Liporto, Jillian L. Joyce, Michael Ryan Kann, Stephanie Cosentino, Preeti Sunderaraman

**Affiliations:** 1 Taub Institute for Research on Alzheimer’s Disease and the Aging Brain, Columbia University Irving Medical Center, New York, NY 10032, USA; leah.waltrip@du.edu (L.H.W.); sc4056@cumc.columbia.edu (S.C.); sc2460@cumc.columbia.edu (S.C.); 2 Department of Neurology, Columbia University Irving Medical Center, New York, NY 10032, USA; 3 Department of Psychology, University of Denver, Denver, CO 80210, USA; 4 Department of Neurology, Chobanian & Avedisian School of Medicine, Boston University, Boston, MA 02218, USA; mabouchard@carene.org; 5 Leonard Davis School of Gerontology, University of Southern California, Los Angeles, CA 90033, USA; 6 Gertrude H. Sergievsky Center, Columbia University Irving Medical Center, New York, NY 10032, USA; 7 School of Medicine, University of Pittsburgh, Pittsburgh, PA 15213, USA; kann.michael@medstudent.pitt.edu; 8 Memory & Aging Program at Butler Hospital, Providence, RI 02906, USA; 9 Department of Psychiatry and Human Behavior, Brown University, Providence, RI 02912, USA

**Keywords:** risk-taking, aging, cognitive abilities, domain-specific, finance

## Abstract

**Background/Objectives**: Risk taking, a crucial component of decision-making, is domain-specific. However, most literature has focused on financial risk-taking in relation to cognitive functioning. The current study investigated the association between risk-taking behaviors in five different domains and various cognitive abilities in cognitively normal older adults. **Methods**: Participants (mean age = 69.55 ± 7.35 years; mean education = 16.69 ± 2.19 years; 58.9% female) completed the Domain-Specific Risk-Taking Scale (DOSPERT), consisting of financial, health, ethical, recreational, and social risk-taking questions. Cognitive performance on associative memory, verbal memory, working memory, verbal fluency, processing speed, and executive function was examined. Linear regression models adjusted for age, gender, and education level were conducted. **Results**: Two out of five risk-taking domains were associated with various aspects of cognition. **Conclusions**: Financial risk-aversion was linked to better memory, while health and safety risk-taking was linked to faster processing speed. These findings have practical implications in the context of everyday decision making.

## 1. Introduction

Cognitive aging impacts the way individuals process and make different types of decisions, such as those related to investing [1] and risk-taking [2,3]. Evidence further indicates that pathological cognitive changes in the brain can uniquely impact different decision-making domains. For example, older adults with Alzheimer’s disease (AD) demonstrate increased risk taking in medical-decision making and financial decisions with monetary rewards [4], while those with behavioral variants of frontotemporal dementia show deficits in reward-based information processing, wherein there is an increase in risk-taking behaviors such as pathological gambling [5]. Considering that the progression of various neurocognitive diseases is associated with increased risk-taking in daily life (i.e., driving when visually impaired, walking without assistive devices) [6], assessing the cognitive correlates of risk-taking in cognitively normal older adults is an important initial step in understanding and reducing the potentially dangerous consequences associated with risky decision-making among older adults.

However, the current literature assessing risk-taking behavior in older adults has limitations. Some studies have employed measures of risk-taking which have not been validated [7]. Additionally, most studies have enrolled samples which may consist of older adults with and without cognitive impairments, making it difficult to determine whether the observed differences occur in the context of neurodegenerative conditions or typical aging [1,8,9,10]. Moreover, several studies recruited older adults from senior housing facilities, and it is unclear whether these individuals were fully independent in their decision making or whether they received assistance in this regard [1,8,9,10,11]. It is therefore essential to comprehensively examine the associations between risk-taking and various cognitive abilities in cognitively normal older adults to identify risk-taking profiles. Then when individuals show cognitive impairments and deviate from expected risk-taking profiles, focused interventions can be devised to prevent potentially adverse consequences.

Risk-taking is highly-domain-specific and context-dependent [12,13], and there is evidence that the pattern of financial risk-taking across the lifespan is distinct from risk-taking in social, health, and ethical domains [8]. Nonetheless, the current literature linking risk-taking and cognitive functioning in older adults has been limited and has often only included a single domain of risk-taking: financial. Studies utilizing financial risk-taking tasks have reported that risk-aversion (the tendency to “play it safe” to a fault) is associated with worse cognitive outcomes [14,15,16,17]. Specifically, global cognitive functioning [14,16], as well as semantic memory, episodic memory, working memory, and perceptual speed have been negatively associated with financial risk aversion [16]. While financial risk-taking is one important domain of daily decision-making, previous work has revealed that risk-taking is highly domain-specific, indicating that one’s propensity to engage in financial risk-taking may not be related to other decisions made in daily life regarding health, social interactions, or recreational activities [12,13]. Indeed, a cross-sectional study of risk-taking across the adult lifespan by Rolison and colleagues established that older adults’ financial risk-taking behavior does not relate to risk taking in other domains of life [8]. Though some studies assessing risk-taking in older adults have employed measures that allow for multiple dimensions of risk-taking to be assessed [8,9], these studies have not tested the association of different domains of risk-taking and cognitive function, instead focusing on the effects of age [8] and mood [8] on risk-taking. Given the research body establishing risk-taking as context-dependent, it is therefore important to study the link between multiple domains of risk-taking and cognitive function in older adults.

In the present study, we aimed to examine the association between risk-taking in five specific domains (financial, health and safety, social, ethical, and recreational) and various cognitive abilities (processing speed, working memory, phonemic fluency, semantic fluency, episodic memory, and associative memory) in older adults rigorously characterized as cognitively healthy. We hypothesized that general risk-taking would not be significantly associated with any cognitive outcomes due to its non-specific nature. Unlike domain-specific measures of risk-taking which capture risk-taking likelihood within clearly defined contexts, general risk-taking aggregates across multiple domains. This aggregation can obscure potential associations by blending risk-taking tendencies that can be explained by different motivations or cognitive reasoning. As a result, we expected that general risk-taking would lack the specificity required to align with particular cognitive domains, leading to null associations in our analyses. Based on the existing literature linking risk-taking and cognition in older adults [15,16], we hypothesized that financial risk-taking would be associated with episodic and working memory, such that those who were financially risk-averse would perform worse on cognitive measures. Additionally, we performed exploratory analyses on the relationships between health and safety, social, ethical and recreational risk-taking domains and their relationship to the aforementioned cognitive abilities. We also performed exploratory analyses on sub-domains of financial risk-taking (investment and gambling). These analyses allowed us to probe whether domain-specific patterns of risk-taking, beyond financial risk, might demonstrate distinct associations with cognition, given that each domain reflects unique motivational drivers and behavioral tendencies.

## 2. Materials and Methods

### 2.1. Participants

Fifty-six cognitively healthy older adults were eligible for this study. Participants were recruited from the Columbia University Irving Medical Center Department of Neurology Memory Disorders Clinic, the Alzheimer’s Disease Research Center at Columbia University, and the Cognitive Reserve and Reference Ability Neural Network (CR/RANN) studies at Columbia University Department of Neurology [18]. Participants were on average 69.55 years old (±7.35), highly educated (16.69 ± 2.19 years of education), and 33 out of 56 participants (58.9%) were female. Participants were majority White (80.4%; 10.7% Black; 5.4% Asian; 3.6% chose not to identify) and non-Hispanic (96.4%). Data was collected in New York City between May of 2016 and January of 2018.

For inclusion in the study, participants had to score within clinically normal limits (≥−1.5 SD based on demographically adjusted normative data) on standardized tests evaluating memory, executive function, and language (described below in Section 2.2). Participants with any history of neurological conditions (e.g., stroke, traumatic brain injury, brain tumors, etc.) or major psychiatric disorders (e.g., bipolar disorder and schizophrenia) were excluded from the study after medical chart review and intake interviews. For more details, please see Chapman et al., 2021 [19]. Participants were consented with written consent approved by the Institutional Review Board at Columbia University. Data was collected in New York City between May of 2016 and January of 2018.

### 2.2. Measures

#### Risk-Taking

Risk-taking was assessed using the Domain-Specific Risk-Taking (DOSPERT) Scale [12]. The validated, 30-item, self-report survey, is a revised version of the Weber et al., 2002 DOSPERT Scale [13], which was devised to assess risk perceptions and risk behaviors in five content domains: financial, health and safety, social, ethical, and recreational. Additionally, the financial domain can be broken down into two subdomains labeled gambling and investing. In Part I of this scale, participants are asked to rate the likelihood of their engagement in domain-specific risky behaviors. They are instructed to “Indicate the likelihood that (they) would engage in the described activities and behaviors if (they) were to find (themselves) in that situation” and provided with a 7-point Likert scale wherein 1 is equivalent to “Extremely Unlikely” and 7 is equivalent to “Extremely Likely” so that higher scores indicate higher likelihood of risk-taking. Please see Table 1 for a list of all items within each domain. This scale demonstrates acceptable internal consistency estimates (Cronbach’s Alpha between 0.71 and 0.86) [12].

### 2.3. Cognitive Tests

All participants were administered a comprehensive battery of cognitive tests assessing five domains including working memory, processing speed, phonemic fluency, semantic fluency, and verbal memory.

i Processing Speed and Working Memory: The Trail Making Test is a measure of attention, processing speed, and mental flexibility where participants are asked to connect circles on a page as fast as possible without lifting their pencil. Part A, wherein participants are required to connect 25 randomly placed numbers on a page in numerical order, was used to assess processing speed. Part B, wherein examinees are asked to alternate between connecting numbers and alphabetical letters, was used to measure working memory [20]. On the Trail Making Test, score is dictated by seconds for completion, with higher scores indicating a slower/worse performance.
ii Phonemic Fluency: Phonemic fluency was assessed using the ‘CFL’ version of the Controlled Oral Word Association Test, where participants are asked to recite from memory as many words that they can which begin with ‘c’, f’, or ‘l’ within a 60 s time limit [21]. Higher scores are indicative of more words being recited, so higher scores represent better performance on the CFL Task.
iii Semantic Fluency: Semantic fluency was assessed using the ‘Animal Naming’ version of the category verbal fluency test. Participants are asked to name as many animals as they can think of within 60 s, so higher scores are indicative of better performance on the category verbal fluency test.
iv Verbal Memory: Verbal memory was assessed with the Selective Reminding task (SRT), which includes both an immediate recall and delayed recall trial. The SRT is a cued verbal list-learning task consisting of 6 trials, with 12 words in each trial [22]. The SRT is used to detect episodic memory impairment in clinical and research settings at Columbia University where participants for the study were recruited. Higher scores are indicative of more word recall.
v Associative Memory: Associative memory was assessed using the Face-Name Associative Memory Task (FNAME), which requires participants to learn names and occupations associated with faces shown to them [23,24]. The FNAME consists of a learning trial, an immediate memory trial, and a delayed recall trial and higher scores indicate better performance on the task. Immediate and delayed recall scores for the naming portion of the task were utilized for this study. The names portion of the FNAME was chosen for this study as performance on the naming portion has been linked to amyloid burden in healthy older adults, indicating it may be a cognitive measure of preclinical AD in aging populations [23]. This approach has been used before with similar populations [19].

All cognitive tests, except for the FNAME, were conducted as a part of study screening for participants. The DOSPERT and FNAME were administered within one year of cognitive screening tests.

### 2.4. Statistical Analyses

Statistical analyses were conducted with IBM SPSS Statistics Version 27 [25]. Data were assessed for normality, outliers, and homoscedasticity. Two participants whose SRT administration was more than one year from the administration of the DOSPERT were removed from analyses involving the SRT.

A series of linear regression models were run to examine whether general (sum score of DOSPERT measure) and domain-specific risk-taking were associated with cognitive test scores. Exploratory analyses on the investment and gambling sub-domains were also conducted. The models were adjusted for demographic variables known to be significantly associated with risk-taking and cognition including age, gender, and years of education [26,27,28]. Models were additionally adjusted for time between assessments where applicable. Finally, Pearsons correlations among the DOSPERT domains were conducted to examine the intercorrelations among the five domains.

## 3. Results

Demographic information, as well as a breakdown of the range, mean, and standard deviation of the DOSPERT subscales and all cognitive tests are summarized in Table 2. Results of multiple linear-regressions with risk-taking scores as predictors and each cognitive score as the outcome adjusted for age, years of education, gender, and time between assessments in days (where applicable) are shown in Supplementary Tables S1–S8. The intercorrelations among the DOSPERT domains are reflected in Supplementary Table S9. The financial risk-taking domain was significantly associated with SRT delayed recall performance (R^2^ = 0.457, F (5, 48) = 2.535, *p* = 0.041), FNAME immediate names recall (R^2^ = 0.212, F (4, 50) = 3.355, *p* = 0.016), and FNAME delayed names recall (R^2^ = 0.175, F (4, 50) = 2.644, *p* = 0.044) such that higher memory scores were associated with lower endorsement of financial risk-taking. Financial risk-taking was the only significant predictor of each model (SRT Delayed: β = −0.342, *p* = 0.012; FNAME immediate name recall: β = −0.313, *p* = 0.019; FNAME delayed name recall: β = −0.298, *p* = 0.028). Additionally, exploratory analyses showed that the investment sub-domain of financial risk-taking was significantly associated with FNAME immediate names recall such that higher scores were associated with lower endorsement of investment risk-taking (R^2^ = 0.192, F (4, 50) = 2.979, *p* = 0.028), with investment risk-taking being the only significant predictor of the model (β = −0.278, *p* = 0.037).

In addition, the health and safety risk-taking domain was significantly associated with Trails A task performance such that faster processing speed was associated with higher endorsement of health and safety risk-taking (R^2^ = 0.205, F (5, 48) = 2.475, *p* = 0.045) wherein health and safety risk-taking was the only significant predictor (β = −0.299, *p* = 0.029). No other domain-specific risk-taking associations with cognitive abilities were found.

## 4. Discussion

The current exploratory study examined the associations between risk-taking in five specific domains and various aspects of cognition in a well-characterized group of older adults without cognitive impairments. As hypothesized, we found that general or non-domain-specific risk-taking was not associated with cognitive abilities. In contrast, two out of five specific domains of risk-taking were uniquely associated with cognitive abilities, including various aspects of memory and processing speed. Our findings support a large body of literature, which has shown that financial risk-taking behavior is associated with cognitive abilities [14,15,16] and that risk-taking behavior is domain-specific [8,29]. To our knowledge, we are the first study to show that the relationship between cognitive abilities and risk-taking behaviors may differ in a domain-wise manner in cognitively normal older adults.

### 4.1. Financial Risk-Taking and Memory Tasks

The strongest associations were found for financial risk taking, specifically its association with memory tasks. Previous research has found financial risk aversion to be associated with worse cognitive performance, specifically on measures of working memory, episodic verbal memory, semantic memory, and perceptual speed [14,15,16,17]. We found the opposite trend with financial risk-aversion (the tendency to avoid taking financial risks) being associated with *better* episodic and associative memory. The discrepant findings might be accounted for by cohort and age effects. Specifically, in the Boyle et al.’s [16] and James et al.’s [14] studies, participants were older by almost two decades compared to the current study. The attitudes, perceptions, and abilities of individuals might vary across age and the timeframe in which the study is conducted. In their study, moreover, a different set of instruments were used to measure risk aversion. Their study used a set of questions from the behavioral economic literature which required objective answers (e.g., Would you prefer $15 for sure, OR a coin toss in which you will get $[an amount greater than $15] if you flip heads or nothing if you flip tails?) compared to self-report items used in the current study. Although not measured in the current study, differences in contextual experiences, living conditions (such as marital status, level of support available), perception of riskiness of a situation, and aspects such as level of financial literacy can influence the relationship between cognitive ability and risk aversion.

Our exploratory analyses revealed that the results were driven by the ‘investment’ portion of the financial risk-taking domain. Specifically, better performance on an associative memory task was associated with investment risk-aversion. Conversely, none of the cognitive abilities were related to the gambling portion of financial risk-taking.

One potential explanation for the link between investment behaviors and memory is that both may share a similar underlying neural substrate. The financial domain of risk-taking, especially relating to the investing portion, is the domain of the DOSPERT task in which knowledge about the stock market and topics such as moderate growth mutual funds is necessary to make an informed (non-risky) decision. Therefore, the relatively strong association of memory with investing but not with other cognitive domains seems pragmatic.

Alternatively, these results could reflect the fact that the investing portion is likely the most relevant domain within the DOSPERT for older adults. Supplementary Materials examining the intercorrelations among the DOSPERT domains reveal that the financial domain was not found to be related to other DOSPERT domains (see Supplementary Materials Table S9), further underscoring the uniqueness of this domain.

The DOSPERT was originally developed and validated in a population of undergraduate students whose ages ranged from 16 to 46, with the median age being 18 [13]. While this scale has been used previously to investigate domain-specific risk-taking in older adults [8], the original DOSPERT was designed to capture risk-taking behaviors that are most aligned with a younger population. However, due to a lack of measures which comprehensively assess domain-specificity in risk-taking, use of the DOSPERT for understanding domain-specificity in risk-taking across different age groups is common [30,31]. Therefore, in the health and safety, social, and recreational sub-portions of this survey, there are items which do not align directly with the stage of life that older adults are in. For example, in the social domain, the items “Choosing a career that you truly enjoy over a more prestigious one” and “Starting a new career in your mid-thirties” are questions that require older adults to surmise the riskiness of these actions after they have already lived through them. Older adults, whose life experiences have been much more extensive than younger adults, are likely to answer these questions differently compared to a college student. These findings underscore the utility of age-adapted risk-taking measures.

On the other hand, the investment portion relies on knowledge that some college students may not have had exposure to (mutual funds, stock investment), while older adults may be particularly able to relate to these items. Therefore, it is possible that financial risk-taking is the category which is associated with older adults’ cognitive function because this domain of the DOSPERT more closely relates to older adults’ day-to-day experiences. This association may also be explained by the demographic group involved in this study, as participants were highly educated. It is worth noting that not all older adults are likely to be familiar with terms such as mutual funds and stock investment, and that this may be an artifact of the highly educated nature of participants in our study.

### 4.2. Investment Versus Gambling: Differential Findings and Implications for the Field

The separation in results observed between the investment and gambling portion of the DOSPERT may also be due to the underlying differences in the risk-reward payoff associated with each activity. For most, gambling is a recreational activity with immediate reward payoffs or losses, a pattern of reward which can lead to addiction [32]. In contrast, investing often has no component of immediacy as it can take many months or years to see profit on an investment made. While many adults do not rely on gambling for their financial future, investment is seen as a crucial component of financial management after retirement, when individuals no longer have an income to rely on for expenses.

As such, these findings are particularly relevant as the current literature has relied entirely on gambling adjacent behavioral economics tasks, such as discounting tasks where the choice of a money payoff in the immediate situation potentially yields smaller benefits than those willing to delay or defer their choice [14,15,16].These results suggest that studying risk-taking solely by utilizing tests that mimic gambling may cause researchers to miss a critical aspect of financial risk-taking—investment. Future studies should investigate whether investment risk-taking, above other forms of financial risk-taking, may be a unique predictor of cognitive decline. If supported by future research, this could serve as an important marker of cognitive decline as financial decision making in the realm of investing has the strong potential for negative outcomes if risks are made by those with failing mental capacities [33,34]. Alternatively, memory decline may signal the need for heightened concerns about investment activity for older adults.

### 4.3. Health and Safety Risk-Taking and Processing Speed

Regarding other DOSPERT domains, associations with cognitive abilities were limited to the link between increased health and safety risk-seeking and faster processing speed. One interpretation of this association is that individuals inclined towards risk-taking in this realm might prioritize quick performance. Though faster processing speed can be advantageous, it may also lead to a tendency to prioritize speed over caution, increasing the likelihood of accidents or injuries. As there seems to be an association between fast processing speed and a propensity to take more health and safety related risks, older adults with quick processing speed may be at risk for making quick or impulsive decisions regarding their health and safety which may endanger them (i.e., hurrying through tasks that require careful attention, driving safely, or engaging in physical activities without fully considering the potential dangers). Understanding the relationship between health and safety risk-taking and processing speed is essential for developing strategies that balance the benefits of quick processing speed with the need for heightened safety awareness in older adults, ultimately promoting their well-being and reducing risk. In summary, the specific link between health and safety risk-taking and cognitive function warrants further exploration by studies given that the implications of health and safety risk-taking behaviors in relation to processing speed may be critical for older adults. The health and safety subdomain was most strongly associated with the ethical domain (see Supplementary Materials Table S9) such that higher risk endorsement in one domain was related to higher endorsement in the other domain. The ethical portion of the task includes items which may be more relevant to older adults than to college students (i.e., taking questionable deductions on income tax return, having an affair with a married man/woman, and leaving your young children [or grandchildren] at home while running an errand). The concordance in ratings between the two domains reflects that older adults perceive ethical and health social issues to be closely aligned.

### 4.4. Strengths

This study had multiple strengths. It employed a well-established measure of domain-specific risk-taking, the DOSPERT [12,17,35]. Furthermore, these results were obtained within a population of individuals who were deemed to be cognitively normal using strict inclusion and exclusion criterion, assuring that results are not skewed by adults with cognitive impairment. The fact that links between risk taking and cognition were detected among cognitively normal adults suggests that such links may be even more pronounced among individuals with cognitive impairment. However, to study the relationship between risk-taking and cognitive decline in adults, future work will need to establish causality through longitudinal studies.

### 4.5. Limitations

The present study has a few limitations. First, the sample size is relatively small, limiting the statistical power of this study. Moreover, we conducted a relatively high number of analyses given the exploratory nature of the paper. Despite these analytic limitations, several of the identified associations between financial risk taking and memory suggest moderate effect sizes. Second, the study sample was limited in its ethnic, racial, and educational diversity. This limits the generalizability of the study findings to broader populations. This lack of diversity is a critical consideration, and future studies should recruit more diverse cohorts of older adults when studying risk-taking in association with cognition. Third, the present study did not measure cognitive abilities such as inhibition, problem solving and abstract reasoning which may also be relevant to decision-making. Future research should include such measures of executive function given their potential role in risk-taking and decision making. Moreover, future studies should examine the relationship between risk taking likelihood and risk perception. The perception of risk can be subjective, and this might influence the extent to which one might engage in a specific behavior. Fourth, in our analysis, corrections for multiple comparisons were not utilized and the possibility of Type I error cannot be ruled out. Additionally, this was a cross-sectional study, and causal explanations cannot be provided because it is likely that risk-taking behaviors share a bidirectional relationship with cognitive abilities. Future studies should replicate these findings using more robust analytic approaches and longitudinal studies of cognition in relation to risk-taking should be undertaken.

Overall, our findings highlight the importance of developing a domain-specific risk-taking scale for older adults that is relevant to their daily lived experiences. We chose to use the DOSPERT as it is a validated and well-established measure of domain-specific risk-taking; however, the DOSPERT was originally studied and normed in young adults. Though previous studies have utilized the DOSPERT when studying older adults [8], a measure that is explicitly designed to study domain-specific risk-taking in older adults would be an asset to the field.

### 4.6. Future Directions

In relation to risk-taking, future studies may assess other aspects of financial abilities such as financial literacy and novel simulated online financial decision-making measures such as the Online Money Management Task [36,37]. Comparing the DOSPERT to other previously employed measures of financial risk-taking such as the Balloon Analogue Risk Task and standard behavioral economics surveys previously used [14,16,17] might reveal whether these risk-taking tasks are associated similarly. Lastly, multiple measures of risk-taking likelihood and risk perception, such as behavioral economics tasks and self-report measures, should be employed in such studies to improve confidence in the results.

## 5. Conclusions

The present study reflects the importance of domain-specificity when studying the association of risk-taking to cognition in older adults. Our results indicate that financial risk-taking is strongly related to memory in cognitively normal older adults and highlight the importance of examining financial risk-taking, especially in investments, alongside cognitive memory tests. However, the unexpected direction of our findings compared to the larger literature requires careful attention as our study used a different measure of risk-taking and we found differing results for investing decisions versus gambling decisions within the financial domain. Our results also point to interesting lines for future research implicating the links between cognition and health and safety domain of risk-taking.

## Figures and Tables

**Table 1:** DOSPERT Domain Specific Risk-Taking Scale.

Domain Subscale		Statement	
Financial	Investing	Investing 5% of your annual income in a very speculative stock.	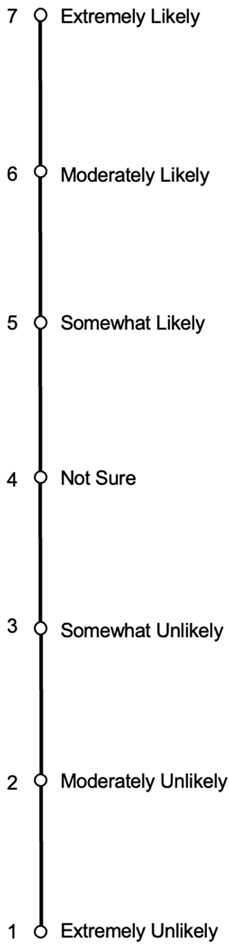
		Investing 10% of your annual income in a moderate-growth mutual fund.	
		Investing 10% of your annual income in a new business venture.	
	Gambling	Betting a day’s income at the horse races.	
		Betting a day’s income on the outcome of a sporting event.	
		Betting a day’s income at a high-stake poker game.	
Total Subscale Score		6–42	
Health/Safety		Drinking heavily at a social function.	
		Engaging in unprotected sex.	
		Driving a car without wearing a seat belt.	
		Riding a motorcycle without a helmet.	
		Sunbathing without sunscreen.	
		Walking home alone at night in an unsafe area of town.	
Total Subscale Score		6–42	
Social		Admitting that your tastes are different from those of a friend.	
		Disagreeing with an authority figure on a major issue.	
		Choosing a career that you truly enjoy over a more prestigious one.	
		Speaking your mind about an unpopular issue in a meeting at work.	
		Moving to a city far away from your extended family.	
		Starting a new career in your mid-thirties.	
Total Subscale Score		6–42	
Ethical		Taking some questionable deductions on your income tax return	
		Having an affair with a married man/woman	
		Passing off somebody else’s work as your own.	
		Revealing a friend’s secret to someone else.	
		Leaving your young children alone at home while running an errand.	
		Not returning a wallet you found that contains $200.	
Total Subscale Score		6–42	
Recreational		Going camping in the wilderness.	
		Going down a ski run that is beyond your ability.	
		Going whitewater rafting at high water in the spring.	
		Taking a skydiving class.	
		Bungee jumping off a tall bridge.	
		Piloting a small plane.	
Total Subscale Score		6–42	
Overall Scale Score (Non-Domain Specific Risk-Taking)		30–210	

**Table 2:** Summary of demographic characteristics, DOSPERT scores, and cognitive test performance.

Variable	N (%) or Mean ± SD (Range)
Female	33 (58.9%)
Age (years)	69.55 ± 7.35 (54–90)
Education (years)	16.69 ± 2.19 (12–20)
Race	
White	45 (80.4%)
Black	6 (10.7%)
Asian	3 (5.4%)
Chose not to identify	2 (3.6%)
Ethnicity	
Non-Hispanic	54 (96.4%)
DOSPERT	
General risk-taking	84.05 ± 17.28 (50–119)
Financial risk-taking	12.93 ± 4.67 (6–24)
Investing risk-taking	9.27 ± 3.99 (3–18)
Gambling risk-taking	3.66 ± 1.64 (3–11)
Health and Safety risk-taking	13.63 ± 5.04 (6–23)
Social risk-taking	31.71 ± 5.98 (19–42)
Ethical risk-taking	11.12 ± 4.24 (6–21)
Recreational risk-taking	14.68 ± 8.11 (6–33)
Cognitive Tests	
Trails A *	29.71 ± 9.28 (11–61)
Trails B *	68.66 ± 25.05 (32–172)
Animal Naming	24.02 ± 4.93 (15–37)
CFL Verbal Fluency	51.00 ± 11.44 (25–77)
SRT Immediate Recall	51.57 ± 7.50 (34–68)
SRT Delayed Recall	8.46 ± 2.18 (4–12)
FaceName Immediate Recall	5.73 ± 3.79 (0–16)
FaceName Delayed Recall	5.68 ± 3.69 (0–15)

N = 56. Values represent the number of patients (%) or mean ± SD (range). * Measured in seconds; higher scores indicate worse performance on these tasks.

## Data Availability

We do not have permission by the IRB to release data in the public domain. However, data and analytic code are available upon request. All materials have original citations and measures that are not under copyright have been provided.

## Supplementary Materials

The following supporting information can be downloaded at https://www.mdpi.com/article/10.3390/brainsci15101044/s1. Table S1. Multivariate Regression Analysis with General Risk Taking; Table S2. Multivariate Regression Analysis with Financial Risk Taking; Table S3. Multivariate Regression Analysis with Investment Risk Taking; Table S4. Multivariate Regression Analysis with Gambling Risk Taking; Table S5. Multivariate Regression Analysis with Health and Safety Risk Taking; Table S6. Multivariate Regression Analysis with Social Risk Taking; Table S7. Multivariate Regression Analysis with Ethical Risk Taking; Table S8. Multivariate Regression Analysis with Recreational Risk Taking. Table S9. Correlations Between DOSPERT Domains.
