# Supplementary material for: Relating Domain-Specific Risk-Taking Behavior to Cognitive Functions in Older Adults
Source: Brain Sci. 2025 Sep 25;15(10):1044. doi: 10.3390/brainsci15101044 (PMC12562380; doi:10.3390/brainsci15101044)
Supplement: Supplementary file 1 [file brainsci-15-01044-s001.zip › brainsci-3846110-supplementary.pdf]

Supplemental Material

Table S1. Multivariate Regression Analysis with General Risk Taking

	Dependent Variable	Independent Variables	Beta	P-Value
General Risk Taking	Trails A	General risk-taking	-0.213	0.137
		Age	0.248	0.106
		Education	-0.170	0.267
		Gender	-0.033	0.817
		Time between assessments (days)	-0.200	0.194
		Model Summary: $R^2 = 0.161$, $F(5, 48) = 1.843$, $p = 0.122$		
	Trails B	General risk-taking	-0.125	0.353
		Age	0.426	0.006**
		Education	-0.229	0.120
		Gender	-0.024	0.860
		Time between assessments (days)	0.032	0.828
		Model Summary: $R^2 = 0.182$, $F(5, 49) = 2.174$, $p = 0.072$		
	CFL	General risk-taking	-0.038	0.783
		Age	-0.085	0.578
		Education	0.314	0.041*
		Gender	-0.118	0.399
		Time between assessments (days)	-0.081	0.602
		Model Summary: $R^2 = 0.134$, $F(5, 48) = 1.480$, $p = 0.214$		
	Animal Naming	General risk-taking	0.139	0.335
		Age	-0.191	0.261
		Education	-0.043	0.792
		Gender	0.180	0.219
		Time between assessments (days)	0.078	0.616
		Model Summary: $R^2 = 0.128$, $F(5, 46) = 1.355$, $p = 0.259$		
	SRT Immediate Recall	General risk-taking	0.039	0.778
		Age	-0.392	0.020*
		Education	-0.126	0.394
		Gender	0.071	0.613
		Time between assessments (days)	-0.345	0.040*
		Model Summary: $R^2 = 0.153$, $F(5, 47) = 1.695$, $p = 0.154$		
	SRT Delayed Recall	General risk-taking	-0.118	0.398
		Age	-0.276	0.101
		Education	-0.191	0.206
		Gender	0.078	0.579
		Time between assessments (days)	-0.223	0.183
		Model Summary: $R^2 = 0.131$, $F(5, 47) = 1.416$, $p = 0.236$		
	FaceName Immediate Recall	General risk-taking	-0.045	0.740
		Age	-0.276	0.057
		Education	0.227	0.121
		Gender	0.173	0.212
		Model Summary: $R^2 = 0.107$, $F(4, 51) = 1.526$, $p = 0.208$		
	FaceName Delayed Recall	General risk-taking	-0.077	0.574
		Age	-0.264	0.071
		Education	0.141	0.335
		Gender	0.174	0.213
		Model Summary: $R^2 = 0.097$, $F(4, 51) = 1.364$, $p = 0.259$		

*Statistically significant p<0.05
**Statistically significant p<0.01

Supplementary Table S2. Multivariate Regression Analysis with Financial Risk Taking

	Dependent Variable	Independent Variables	Beta	P-Value
Financial Risk Taking	Trails A	Financial risk-taking	-0.037	0.794
		Age	0.287	0.065
		Education	-0.143	0.374
		Gender	0.020	0.890
		Time between assessments (days)	-0.155	0.315
		Model Summary: R^2 = 0.122, F (5, 48) = 1.338, p = 0.265		
	Trails B	Financial risk-taking	0.065	0.628
		Age	0.446	0.004**
		Education	-0.207	0.171
		Gender	-0.005	0.970
		Time between assessments (days)	0.061	0.674
		Model Summary: R^2 = 0.171, F (5, 49) = 2.020, p = 0.092		
	CFL	Financial risk-taking	-0.160	0.244
		Age	-0.084	0.575
		Education	0.279	0.070
		Gender	-0.109	0.427
		Time between assessments (days)	-0.093	0.543
		Model Summary: R^2 = 0.157, F (5, 48) = 1.783, p =0.134		
	Animal Naming	Financial risk-taking	0.119	0.391
		Age	-0.315	0.043*
		Education	0.561	0.577
		Gender	0.153	0.272
		Time between assessments (days)	-0.027	0.856
		Model Summary: R^2 = 0.123, F (5, 49) = 1.376, p = 0.249		
	SRT Immediate Recall	Financial risk-taking	-0.090	0.520
		Age	-0.349	0.040*
		Education	-0.079	0.606
		Gender	0.053	0.706
		Time between assessments (days)	-0.285	0.086
		Model Summary: R^2 = 0.121, F (5, 48) = 1.317, p = 0.273		
	SRT Delayed Recall	**Financial risk-taking**	**-0.342**	**0.012***
		Age	-0.228	0.151
		Education	-0.228	0.121
		Gender	0.099	0.453
		Time between assessments (days)	-0.168	0.281
		**Model Summary: R^2 = 0.457, F (5, 48) = 2.535, p = 0.041***		
	FaceName Immediate Recall	**Financial risk-taking**	**-0.313**	**0.019***
		Age	0.260	0.058
		Education	0.222	0.116
		Gender	0.146	0.257
		**Model Summary: R^2 = 0.212, F (4, 50) = 3.355, p = 0.016***		
	FaceName Delayed Recall	**Financial risk-taking**	**-0.298**	**0.028***
		Age	-0.244	0.081
		Education	0.128	0.372
		Gender	0.156	0.239
		**Model Summary: R^2 = 0.175, F (4, 50) = 2.644, p = 0.044***		

*Statistically significant p<0.05
**Statistically significant p<0.01

Supplementary Table S3. Multivariate Regression Analysis with Investment Risk Taking

	Dependent Variable	Independent Variables	Beta	P-Value
Investment Risk Taking	Trails A	Investment risk-taking	-0.025	0.865
		Age	0.287	0.065
		Education	-0.140	0.390
		Gender	0.020	0.890
		Time between assessments (days)	-0.154	0.322
		Model Summary: R^2 = 0.122, F (5, 48) = 1.329, p = 0.268		
	Trails B	Investment risk-taking	0.043	0.753
		Age	0.446	0.004**
		Education	-0.211	0.164
		Gender	-0.028	0.978
		Time between assessments (days)	0.060	0.683
		Model Summary: R^2 = 0.169, F (5, 49) = 1.987, p = 0.097		
	CFL	Investment risk-taking	-0.189	0.170
		Age	-0.087	0.562
		Education	1.810	0.077
		Gender	-0.832	0.410
		Time between assessments (days)	-0.099	0.513
		Model Summary: R^2 = 0.166, F (5, 48) = 1.910, p = 0.110		
	Animal Naming	Investment risk-taking	0.168	0.227
		Age	-0.311	0.044*
		Education	0.100	0.515
		Gender	0.155	0.261
		Time between assessments (days)	-0.017	0.907
		Model Summary: R^2 = 0.136, F (5, 49) = 1.545, p = 0.194		
	SRT Immediate Recall	Investment risk-taking	-0.052	0.709
		Age	-0.353	0.038*
		Education	-0.072	0.639
		Gender	0.051	0.717
		Time between assessments (days)	-0.291	0.079
		Model Summary: R^2 = 0.115, F (5, 48) = 1.253, p = 0.299		
	SRT Delayed Recall	Investment risk-taking	-0.312	0.023*
		Age	-0.239	0.138
		Education	-0.227	0.128
		Gender	0.092	0.492
		Time between assessments (days)	-0.185	0.240
		Model Summary: R^2 = 0.190, F (5, 48) = 2.253, p = 0.064		
	FaceName Immediate Recall	**Investment risk-taking**	**-0.278**	**0.037***
		Age	-0.265	0.056
		Education	0.233	0.104
		Gender	0.149	0.255
		**Model Summary: R^2 = 0.192, F (4, 50) = 2.979, p = 0.028***		
	FaceName Delayed Recall	Investment risk-taking	-0.272	0.046*
		Age	-0.248	0.078
		Education	0.137	0.345
		Gender	0.158	0.237
		Model Summary: R^2 = 0.161, F (4, 50) = 2.390, p = 0.063		

*Statistically significant p<0.05
**Statistically significant p<0.01

Supplementary Table S4. Multivariate Regression Analysis with Gambling Risk Taking

	Dependent Variable	Independent Variables	Beta	P-Value
Gambling Risk Taking	Trails A	Gambling risk-taking	-0.116	0.404
		Age	0.337	0.028*
		Education	-0.248	0.118
		Gender	0.072	0.603
		Time between assessments (days)	-0.227	0.136
		Model Summary: R^2 = 0.195, F (5, 46) = 2.229, p = 0.067		
	Trails B	Gambling risk-taking	0.168	0.204
		Age	0.472	0.002**
		Education	-0.235	0.111
		Gender	0.005	0.969
		Time between assessments (days)	0.036	0.803
		Model Summary: R^2 = 0.199, F (5, 48) = 2.386, p = 0.052		
	CFL	Gambling risk-taking	-0.060	0.662
		Age	-0.102	0.511
		Education	0.325	0.037*
		Gender	-0.120	0.388
		Time between assessments (days)	-0.065	0.673
		Model Summary: R^2 = 0.141, F (5, 47) = 1.539, p = 0.196		
	Animal Naming	Gambling risk-taking	-0.131	0.341
		Age	-2.195	0.033*
		Education	0.067	0.662
		Gender	0.149	0.286
		Time between assessments (days)	-0.028	0.853
		Model Summary: R^2 = 0.128, F (5, 48) = 1.406, p = 0.239		
	SRT Immediate Recall	Gambling risk-taking	-0.116	0.408
		Age	-0.348	0.045*
		Education	-0.063	0.679
		Gender	0.056	0.692
		Time between assessments (days)	-0.275	0.102
		Model Summary: R^2 = 0.120, F (5, 47) = 1.283, p = 0.287		
	SRT Delayed Recall	Gambling risk-taking	-0.256	0.066
		Age	-0.266	0.113
		Education	-0.153	0.306
		Gender	0.089	0.517
		Time between assessments (days)	-0.168	0.303
		Model Summary: R^2 = 0.160, F (5, 47) = 1.793, p = 0.133		
	FaceName Immediate Recall	Gambling risk-taking	-0.221	0.100
		Age	-0.301	0.037*
		Education	0.214	0.140
		Gender	0.152	0.260
		Model Summary: R^2 = 0.154, F (4, 50) = 2.278, p = 0.074		
	FaceName Delayed Recall	Gambling risk-taking	-0.227	0.094
		Age	-0.294	0.043*
		Education	0.132	0.361
		Gender	0.154	0.255
		Model Summary: R^2 = 0.144, F (4, 50) = 2.108, p = 0.094		

*Statistically significant p<0.05
**Statistically significant p<0.01

Supplementary Table S5. Multivariate Regression Analysis with Health and Safety Risk Taking

	Dependent Variable	Independent Variables	Beta	P-Value
Health and Safety Risk Taking	Trails A	**Health/Safety risk-taking**	**-0.299**	**0.029***
		Age	0.243	0.101
		Education	-0.164	0.267
		Gender	-0.028	0.837
		Time between assessments (days)	-0.150	0.304
		**Model Summary: $R^2 = 0.205$, F (5, 48) = 2.475, p = 0.045***		
	Trails B	Health/Safety risk-taking	-0.159	0.232
		Age	0.423	0.006**
		Education	-0.230	0.116
		Gender	-0.023	0.863
		Time between assessments (days)	0.058	0.686
		Model Summary: $R^2 = 0.191$, F (5, 49) = 2.315, p = 0.058		
	CFL	Health/Safety risk-taking	0.113	0.413
		Age	-0.070	0.646
		Education	0.312	0.041*
		Gender	-0.097	0.485
		Time between assessments (days)	-0.085	0.578
		Model Summary: $R^2 = 0.144$, F (5, 48) = 1.619, p = 0.173		
	Animal Naming	Health/Safety risk-taking	-0.038	0.790
		Age	-0.213	0.211
		Education	-0.058	0.725
		Gender	0.146	0.321
		Time between assessments (days)	0.056	0.718
		Model Summary: $R^2 = 0.112$, F (5, 46) = 1.158, p = 0.344		
	SRT Immediate Recall	Health/Safety risk-taking	-0.124	0.382
		Age	-0.358	0.034*
		Education	-0.062	0.687
		Gender	0.032	0.821
		Time between assessments (days)	-0.266	0.112
		Model Summary: $R^2 = 0.127$, F (5, 48) = 1.397, p = 0.242		
	SRT Delayed Recall	Health/Safety risk-taking	-0.063	0.660
		Age	-0.250	0.140
		Education	-0.161	0.293
		Gender	0.081	0.568
		Time between assessments (days)	-0.187	0.267
		Model Summary: $R^2 = 0.101$, F (5, 48) = 1.083, p = 0.382		
	FaceName Immediate Recall	Health/Safety risk-taking	-0.067	0.626
		Age	-0.281	0.054
		Education	0.223	0.129
		Gender	0.170	0.218
		Model Summary: $R^2 = 0.109$, F (4, 51) = 1.562, p = 0.199		
	FaceName Delayed Recall	Health/Safety risk-taking	-0.080	0.563
		Age	-0.268	0.068
		Education	0.137	0.351
		Gender	0.174	0.210
		Model Summary: $R^2 = 0.097$, F (4, 51) = 1.369, p = 0.258		

*Statistically significant p<0.05
**Statistically significant p<0.01

Table S6. Multivariate Regression Analysis with Social Risk Taking

	Dependent Variable	Independent Variables	Beta	P-Value
Social Risk Taking	Trails A	Social risk-taking	-0.223	0.113
		Age	0.253	0.097
		Education	-0.143	0.343
		Gender	-0.016	0.907
		Time between assessments (days)	-0.215	0.167
		Model Summary: R^2 = 0.166, F (5, 48) = 1.913, p = 0.109		
	Trails B	Social risk-taking	-0.168	0.212
		Age	0.422	0.006**
		Education	-0.217	0.137
		Gender	-0.023	0.866
		Time between assessments (days)	0.011	0.942
		Model Summary: R^2 = 0.193, F (5, 49) = 2.346, p = 0.055		
	CFL	Social risk-taking	0.126	0.374
		Age	-0.125	0.424
		Education	0.189	0.220
		Gender	-0.078	0.583
		Time between assessments (days)	0.126	0.376
		Model Summary: R^2 = 0.097, F (5, 49) = 1.057, p = 0.395		
	Animal Naming	Social risk-taking	0.211	0.129
		Age	-0.290	0.059
		Education	0.052	0.725
		Gender	0.179	0.195
		Time between assessments (days)	0.211	0.929
		Model Summary: R^2 = 0.151, F (5, 49) = 1.744, p = 0.142		
	SRT Immediate Recall	Social risk-taking	0.212	0.130
		Age	-0.315	0.060
		Education	-0.071	0.631
		Gender	0.072	0.600
		Time between assessments (days)	-0.236	0.154
		Model Summary: R^2 = 0.155, F (5, 48) = 1.756, p = 0.140		
	SRT Delayed Recall	Social risk-taking	-0.101	0.485
		Age	-0.324	0.061
		Education	-0.099	0.512
		Gender	0.136	0.337
		Time between assessments (days)	-0.338	0.051
		Model Summary: R^2 = 0.136, F (5, 47) = 1.475, p = 0.216		
	FaceName Immediate Recall	Social risk-taking	0.129	0.334
		Age	-0.264	0.066
		Education	0.212	0.147
		Gender	0.187	0.168
		Model Summary: R^2 = 0.121, F (4, 51) = 1.760, p = 0.151		
	FaceName Delayed Recall	Social risk-taking	0.101	0.454
		Age	-0.251	0.084
		Education	0.131	0.372
		Gender	0.192	0.162
		Model Summary: R^2 = 0.101, F (4, 51) = 1.433, p = 0.237		

*Statistically significant p<0.05
**Statistically significant p<0.01

Supplementary Table S7. Multivariate Regression Analysis with Ethical Risk Taking

	Dependent Variable	Independent Variables	Beta	P-Value
Ethical Risk Taking	Trails A	Ethical risk-taking	-0.148	0.283
		Age	0.280	0.068
		Education	-0.152	0.324
		Gender	0.031	0.828
		Time between assessments (days)	-0.171	0.263
		Model Summary: $R^2 = 0.142$, $F (5, 48) = 1.590$, $p = 0.181$		
	Trails B	Ethical risk-taking	-0.173	0.189
		Age	0.438	0.004**
		Education	-0.234	0.109
		Gender	0.016	0.906
		Time between assessments (days)	0.034	0.813
		Model Summary: $R^2 = 0.196$, $F (5, 49) = 2.389$, $p = 0.051$		
	CFL	Ethical risk-taking	0.304	0.027*
		Age	-0.061	0.681
		Education	0.269	0.070
		Gender	-0.117	0.385
		Time between assessments (days)	0.006	0.966
		Model Summary: $R^2 = 0.191$, $F (5, 47) = 2.217$, $p = 0.068$		
	Animal Naming	Ethical risk-taking	0.205	0.144
		Age	-0.211	0.205
		Education	-0.026	0.870
		Gender	0.139	0.327
		Time between assessments (days)	0.074	0.626
		Model Summary: $R^2 = 0.151$, $F (5, 46) = 1.638$, $p = 0.169$		
	SRT Immediate Recall	Ethical risk-taking	-0.124	0.393
		Age	-0.266	0.140
		Education	-0.090	0.555
		Gender	0.110	0.446
		Time between assessments (days)	-0.125	0.478
		Model Summary: $R^2 = 0.099$, $F (5, 47) = 1.029$, $p = 0.412$		
	SRT Delayed Recall	Ethical risk-taking	0.035	0.803
		Age	-0.250	0.141
		Education	-0.159	0.299
		Gender	0.087	0.542
		Time between assessments (days)	-0.205	0.219
		Model Summary: $R^2 = 0.099$, $F (5, 48) = 1.054$, $p = 0.398$		
	FaceName Immediate Recall	Ethical risk-taking	0.044	0.742
		Age	-0.273	0.060
		Education	0.233	0.113
		Gender	0.177	0.196
		Model Summary: $R^2 = 0.107$, $F (4, 51) = 1.526$, $p = 0.209$		
	FaceName Delayed Recall	Ethical risk-taking	0.017	0.901
		Age	-0.258	0.077
		Education	0.146	0.321
		Gender	0.186	0.179
		Model Summary: $R^2 = 0.091$, $F (4, 51) = 1.280$, $p = 0.290$		

*Statistically significant p<0.05
**Statistically significant p<0.01

Supplementary Table S8. Multivariate Regression Analysis with Recreational Risk Taking

	Dependent Variable	Independent Variables	Beta	P-Value
Recreational Risk Taking	Trails A	Recreational risk-taking	0.020	0.889
		Age	0.292	0.062
		Education	-0.131	0.397
		Gender	0.028	0.852
		Time between assessments (days)	-0.147	0.340
		Model Summary: R^2 = 0.121, F (5, 48) = 1.327, p = 0.269		
	Trails B	Recreational risk-taking	0.009	0.948
		Age	0.445	0.004**
		Education	-0.223	0.134
		Gender	-0.001	0.992
		Time between assessments (days)	0.055	0.708
		Model Summary: R^2 = 0.167, F (5, 49) = 1.964, p = 0.101		
	CFL	Recreational risk-taking	-0.266	0.054
		Age	-0.109	0.461
		Education	0.324	0.029*
		Gender	-0.180	0.193
		Time between assessments (days)	-0.102	0.491
		Model Summary: R^2 = 0.197, F (5, 48) = 2.357, p = 0.054		
	Animal Naming	Recreational risk-taking	0.091	0.535
		Age	-0.196	0.253
		Education	-0.065	0.694
		Gender	0.175	0.241
		Time between assessments (days)	0.066	0.669
		Model Summary: R^2 = 0.118, F (5, 46) = 1.229, p = 0.311		
	SRT Immediate Recall	Recreational risk-taking	-0.053	0.715
		Age	-0.283	0.117
		Education	-0.080	0.605
		Gender	0.078	0.596
		Time between assessments (days)	-0.147	0.399
		Model Summary: R^2 = 0.087, F (5, 47) = 0.896, p = 0.492		
	SRT Delayed Recall	Recreational risk-taking	-0.072	0.618
		Age	-0.252	0.137
		Education	-0.154	0.313
		Gender	0.072	0.620
		Time between assessments (days)	-0.200	0.228
		Model Summary: R^2 = 0.102, F (5, 48) = 1.096, p = 0.375		
	FaceName Immediate Recall	Recreational risk-taking	-0.070	0.615
		Age	-0.277	0.056
		Education	0.233	0.111
		Gender	0.162	0.252
		Model Summary: R^2 = 0.109, F (4, 51) = 1.567, p = 0.197		
	FaceName Delayed Recall	Recreational risk-taking	-0.095	0.495
		Age	-0.264	0.070
		Education	0.150	0.304
		Gender	0.161	0.255
		Model Summary: R^2 = 0.099, F (4, 51) = 1.406, p = 0.245		

*Statistically significant p<0.05
**Statistically significant p<0.01

Table S9. Correlations Between DOSPERT Domains

DOPSPERT Domains	1	2	3	4	5
1. Ethical	1	___	___	___	___
2. Financial	0.138	1	___	___	___
3. Health	.484**	0.12	1	___	___
4. Recreation	0.209	0.119	.279*	1	___
5. Social	0.13	0.152	0.212	.267*	1

** Correlation is significant at the 0.01 level (2-tailed).

* Correlation is significant at the 0.05 level (2-tailed).
